# The Impact of Suture Button Removal in Syndesmosis Fixation

**DOI:** 10.3390/jcm10163726

**Published:** 2021-08-21

**Authors:** Jaeyoung Kim, Minsoo Kwon, Jonathan Day, Jesse Seilern und Aspang, Jaehoon Shim, Jaeho Cho

**Affiliations:** 1 Department of Orthopaedic Surgery, Hospital for Special Surgery, New York, NY 10021, USA; jaeyoungkimj@gmail.com; 2 Department of Orthopaedic Surgery, Armed Forces Daejeon Hospital, Daejeon 34059, Korea; msk2050@naver.com; 3 Department of Orthopaedic Surgery, Georgetown University, Washington, DC 20007, USA; jd1553@georgetown.edu; 4 Department of Orthopaedic Surgery, Emory University School of Medicine, Atlanta, GA 30322, USA; jesseseilern@gmail.com; 5 Department of Orthopaedic Surgery, Chuncheon Sacred Heart Hospital, Hallym University, Chuncheon 24253, Korea; shim0121@nate.com

**Keywords:** syndesmosis, syndesmosis injury, suture button, removal, ankle fracture, diastasis, tibiofibular joint

## Abstract

The suture button (SB) device was introduced to negate the need for routine hardware removal in the treatment of syndesmosis injuries. However, a considerable SB removal rate has been reported, and the impact of removal is unknown. This study aimed to evaluate the radiographic and clinical outcomes after removal of SB for syndesmosis fixation. A total of 36 patients who underwent removal surgery after syndesmosis fixation using SB were identified. The mean postoperative time to removal was 12.2 months. On a plain radiograph, tibiofibular clear space (TFCS) was measured and compared at three follow-up time points. In patients with computed tomography (CT) imaging (*n* = 18), the anterior-to-posterior (A/P) ratio was measured to evaluate changes in quality of reduction. Additionally, clinical outcomes were assessed. There were no significant differences in TFCS between the three follow-up periods. None of the patients exhibited recurrent diastasis after SB removal. Although CT analysis demonstrated malreduction in six patients (33.3%), five of six patients had a subsequent spontaneous reduction of the syndesmosis. Clinically, all patients described the resolution of symptoms related to painful hardware at the final follow-up. Our results demonstrate that SB removal at one year following syndesmosis fixation leads to improved clinical symptoms without negatively impacting the quality of syndesmosis reduction.

## 1. Introduction

Syndesmosis injury occurs in 13% of ankle fractures, and one-fifth of all ankle fractures may require surgical fixation [1]. Since these injuries are associated with altered tibiofibular joint kinematics, achieving and maintaining accurate syndesmotic reduction is essential in restoring ankle function and preventing ankle osteoarthritis [2].

Screw fixation has been the most commonly adopted method in the surgical correction of syndesmosis injuries. However, there are ongoing debates over routine screw removal, which is usually performed to prevent screw breakage. There has been evidence to suggest that screw removal is associated with increased cost, infection, and early loss of syndesmosis reduction [3,4,5]. In contrast, more recent computed tomography (CT) studies have demonstrated that screw removal may allow for a spontaneous reduction of a malreduced syndesmosis [6,7]. Consequently, these conflicting results and opinions have raised the need for alternative fixation systems.

To address the shortcomings of screw fixation, implantable suture button (SB) devices were introduced with the advantage of allowing micromotion during healing and potentially eliminating the need for routine hardware removal [8]. There is a growing body of evidence that the SB has similar mechanical strength properties, compared to screws, and yields comparable clinical outcomes [9,10]. However, contrary to its initial design characteristics, a considerable rate of hardware removal has been reported in the literature [11,12,13]. Studies have reported rates of SB removal following syndesmosis fixation as high as 40%, with hardware irritation, infection, osteomyelitis, and osteolysis being the main causes [13,14,15]. However, unlike screw fixation, there is a lack of data evaluating the impact of SB removal on clinical and radiographic outcomes.

The purpose of this study was to investigate the outcomes after SB removal in patients who underwent trans-syndesmotic fixation using SB in acute syndesmosis injuries. We hypothesized that SB removal would not negatively impact radiographic reduction or clinical outcomes.

## 2. Methods

The hospital’s Institutional Review Board approved all aspects of this study protocol (AFMC-16078-IRB-16-067), and informed consent was obtained from all patients.

### 2.1. Subjects

This study retrospectively reviewed medical records of consecutive patients who underwent SB fixation for syndesmotic injuries at our institution between 2009 and 2017. Patients who underwent SB removal and had a radiographic evaluation at three specific time points (immediate postoperative, before SB removal, and at least three months after SB removal) were included in the study cohort. Patients with infection, osteomyelitis, and who underwent revision/reoperation before SB removal were excluded. Ultimately, a total of 33 patients constituted the study cohort. Referring to the previous research on impact to syndesmosis after screw removal [7], the minimum size of samples was analyzed as 28 assuming a significance level of 0.05 and the power of 80%. The mean age of patients was 24.4 (range, 20–35) years. The mean postoperative time to hardware removal was 12.2 (range, 7–19) months, and the mean follow-up time after index surgery was 18.1 (range, 12–36) months. Demographic characteristics and fracture patterns are tabulated in Table 1. 

### 2.2. Surgical Technique

Fixation of the syndesmosis was performed under general or regional anesthesia. In instances of associated ankle fractures, open reduction, and internal fixation (ORIF) to address the respective fracture pattern was performed first. For lateral malleolar (LM) fracture fixation, a locking plate (Arthrex, Naples, FL, USA) that was designed to fit the lateral button of the SB implant was used in most cases; however, different types of plates were used depending on the patients’ anatomy. For medial malleolar (MM) fracture, we used cannulated screw. Additionally, for posterior malleolar (PM) fracture, we used screws and plates for the fracture involving more than 25% of the articular surface, and for a smaller PM fragment, conservative treatment was applied. In cases of isolated syndesmotic injury, a two- or three-hole buttress plate was used with tightrope fixation. The integrity of the syndesmosis was evaluated intraoperatively under fluoroscopy, with an external rotation force applied to the foot or distraction of the fibula from the tibia using a bone hook (Hook test) [16]. When overt separation was observed or overlapping between the tibia and fibula was considered abnormal, SB fixation using the Tightrope^®^ system (Arthrex, Naples, FL, USA) was indicated. A Kirschner guide wire was introduced through the appropriate plate hole or posterior to the plate. After establishing a passage with cannulated drilling, the SB was inserted, tightened, and checked for the maintenance of syndesmotic reduction.

### 2.3. Postoperative Management

Plaster splints were applied in a neutral position after the surgery and removed two weeks postoperatively. Subsequent early range of motion with an ankle brace for six weeks was encouraged. Partial weightbearing with crutches was then permitted as tolerated for patients with isolated syndesmotic injury; patients with additional ORIF for ankle fractures remained non-weightbearing until four weeks postoperatively. Full weightbearing was allowed at six weeks postoperatively, with the progression of activity as tolerated. Full return to sports was permitted after twelve weeks. 

### 2.4. Removal of Suture Button

Removal of the SB was performed when patients presented with symptomatic hardware due to the SB or plates concurrently placed during initial fixation. Removal was conducted using the previous incision with the addition of a stab incision on the medial side under fluoroscopic guidance if needed. In cases where the SB was anchored through a fracture plate hole, concurrent removal of the plate was performed. After removal, patients were allowed to return to previous levels of activity as tolerated.

### 2.5. Radiographic Analysis

On anteroposterior (AP) radiographs of the ankle, tibiofibular clear space (TFCS) was measured electronically with the use of a digital caliper (GE Healthcare, Chicago, IL, USA) with a precision of <0.01 mm. The TFCS was determined as the distance from the incisural surface of the tibia to the medial aspect of the fibula one centimeter proximal to the tibial plafond [17,18,19]. This has been shown in previous studies to be the most reliable point for determining true diastasis with the least dependency on rotational position. The TFCS was measured and compared at three different time points: immediate postoperative (T1), just before SB removal (T2, mean, 12.2 months; range, 7 to 19 months), and at least three months after removal (T3, mean, 5.8 months after removal; range, 3 to 17 months). Our routine practice was to perform a radiographic examination at these follow-up points in patients who have had SB removal to identify potential recurrence after removal. An increase in TFCS of greater than 2mm was considered recurrent diastasis [20,21].

### 2.6. Computed Tomography (CT) Analysis

**Figure 1:**
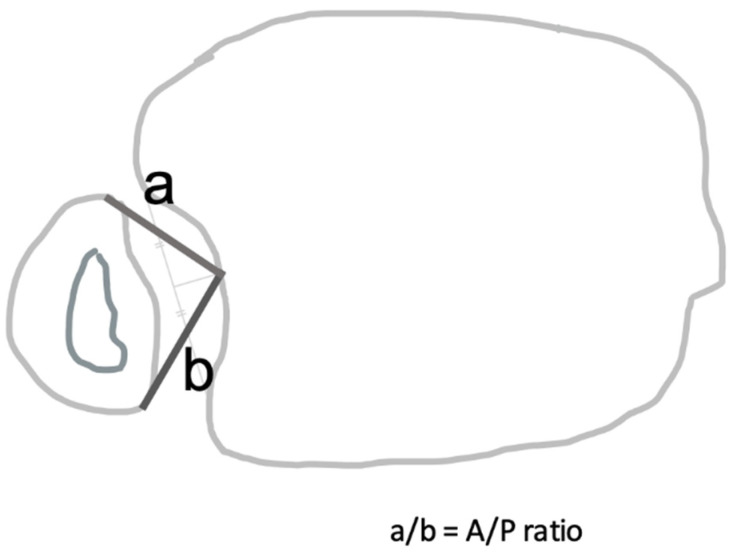
Anteroposterior measurement ratio (A/P ratio) was calculated by measuring the distance from the central point of the incisura to the most anterior (**a**) and posterior (**b**) edge of the fibula. The central point of the incisura was measured by calculating half the distance between the anterior and posterior margins of the incisura.

Within the study cohort, a total of 18 patients were identified to have CT scans at the same follow-up time points (T1, T2, and T3) in the radiology registry. In this group of patients, the accuracy and maintenance of reduction were assessed, as the CT scans would better provide information on the quality of reduction. Single axial images one centimeter proximal to the tibial plafond were investigated. CT images were 3.0 mm cuts [22], and either the third or fourth proximal image from the joint line was used. To maintain a relatively similar anatomic level of images at each follow-up period, the medial and lateral oblong buttons of the SB were identified as landmarks. This approach allowed for accurate reproducibility at the time of measurements. Using a previously defined method by Pelton et al. [23], the anteroposterior measurement ratio (A/P ratio) was calculated (Figure 1). Drawing from the results of previous studies, an A/P ratio between 0.8 and 1.2 was considered normal. In contrast, an A/P ratio greater than 1.2 and less than 0.8 was deemed anteriorly and posteriorly malreduced, respectively [7,23,24]. Measurements at each follow-up period (T1, T2, and T3) were compared.

### 2.7. Clinical Analysis

The resolution of the preoperative symptoms associated with the painful hardware was evaluated at the final follow-up. To assess changes in functional outcome after SB removal, American Orthopedic Foot and Ankle Society (AOFAS) scores and range of ankle motion were evaluated before (T2) and after (T3) SB removal. Any complications related to hardware removal were also evaluated at the final follow-up.

### 2.8. Statistical Analysis

The repeated measure ANOVA test and the generalized estimating equation analysis were used to assess radiographic differences between each follow-up period according to the distributions of categorical variables and continuous variables, respectively. Shapiro–Wilk test was used for normality analysis and paired *t*-tests were used in the analysis of clinical outcomes. Interobserver and intraobserver reliability was determined with the intraclass coefficient (ICC). Two foot and ankle surgeons who were not involved in the surgical procedures performed measurements of radiographic parameters independently on two separate occasions. Measurements were repeated 3 weeks later. Statistical significance was set with an alpha of 0.05. All data were analyzed using “Statistical Package for Social Science (SPSS)” v23.0 for Mac (SPSS Inc., Chicago, IL, USA).

## 3. Results

The indication for hardware removal was pain and irritation arising from the SB and/or plate and screws in all cases. The majority of patients complained of lateral-sided discomfort related to the SB or plate (*n* = 31, 94%), while two patients (6%) complained of medial-sided discomfort associated with the oblong button. 

**Figure 2:**
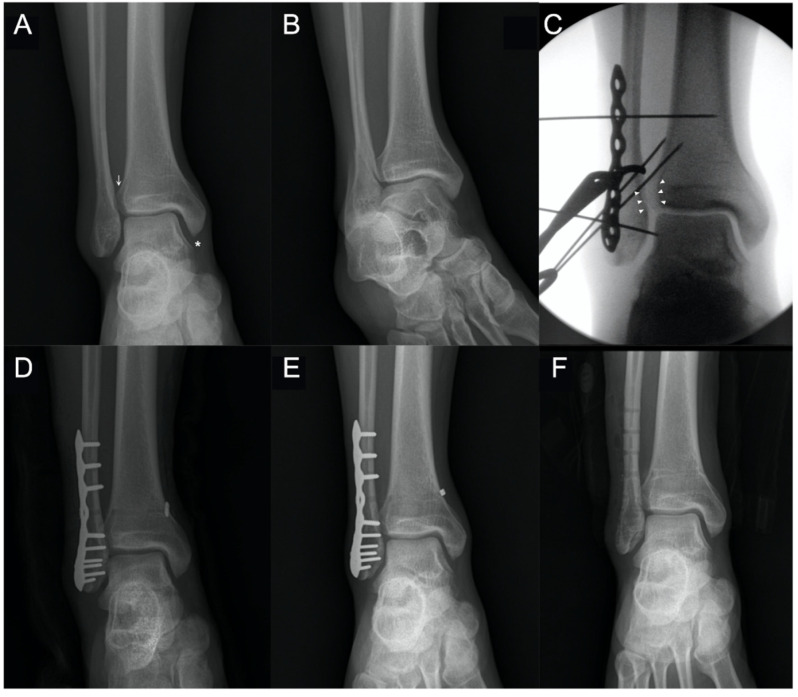
Preoperative, intraoperative, and postoperative images of a patient with syndesmosis injury concurrent with a lateral malleolar fracture: (**A**) ankle anteroposterior (AP) view reveals mild widening tibiofibular space (arrow) and medial clear space (asterisk); (**B**) ankle mortise view shows a fracture line along the fibula; (**C**) intraoperative image demonstrating overt widening of the tibiofibular space (arrowheads), indicating a syndesmosis injury (positive hook test); (**D**–**F**) ankle AP views show maintenance of reduction throughout the follow-up period ((**D**), immediate postoperative; (**E**), just before hardware removal; (**F**), three months after hardware removal).

The TFCS was 4.28 mm, 4.15 mm, and 4.29 mm at T1, T2, and T3, respectively. There were no significant differences between the three follow-up periods. None of the patients exhibited recurrent diastasis following removal of the SB (Figure 2).

**Figure 3:**
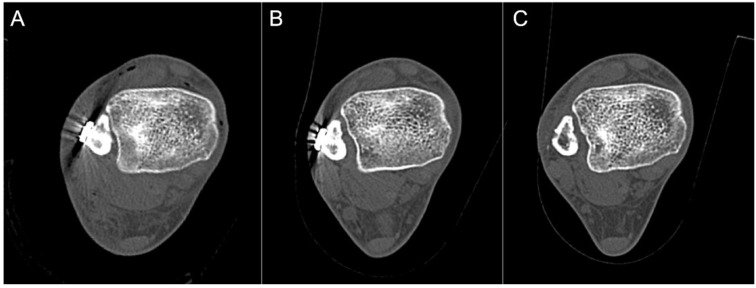
Sequential change of syndesmosis reduction in computed tomography: (**A**) anteriorly malreduced syndesmosis in immediate postoperative (T1) image. (A/P ratio = 1.5); (**B**) spontaneous reduction of malreduced syndesmosis a year after initial fixation (T2, just before hardware removal). Note centered fibular in relation to incisura compared to (**A**) (A/P ratio = 1.2); (**C**) there was no difference in syndesmosis reduction status three months after suture button removal (T3) (A/P ratio = 1.2). Abbreviations: A/P ratio, anteroposterior measurement ratio.

**Figure 4:**
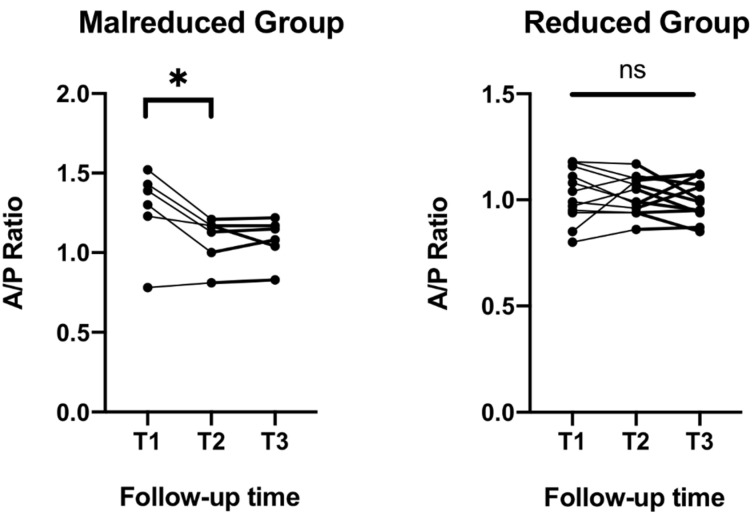
Anteroposterior (A/P) ratio of the syndesmosis in the initially malreduced and adequately reduced group during the study period. In the initially malreduced group, the A/P ratio changed from the immediate postoperative period (T1) to just before suture button removal (T2) with statistical significance (*p* < 0.05), and there were no changes after suture button removal (T3). There were no changes in the adequately reduced group during the study period. *, *p* < 0.05; ns, not significant.

Of the eighteen CT scans performed, six (33.3%) revealed syndesmotic malreduction at T1, with five cases demonstrating anterior malreduction and one demonstrating posterior malreduction. The mean A/P ratio in these six malreduced patients was 1.28 (range, 0.78–1.52). At T2, the mean A/P ratio decreased to 1.08 (range, 0.81–1.21) with statistical improvement (*p* < 0.05). Only one patient remained malreduced, indicating spontaneous resolution of malreduction in five patients, while the SB was retained (Figure 3). The mean A/P ratio at T3 was 1.08 (range, 0.83–1.22), indicating that SB removal did not change the quality of reduction. In patients with adequate initial reduction (*n* = 12), there were no statistically significant differences in the A/P ratio throughout the follow-up period (Figure 4).

Intraobserver reliability was rated excellent with an interobserver correlation coefficient (ICC) of greater than 0.97 for plain radiographic measurements (TFCS), while the A/P ratio in CT analysis revealed good correlation (ICC = 0.86). Interobserver reliability for the TFCS present excellent correlation (ICC = 0.96), while A/P ratio revealed good correlation (ICC = 0.80). 

**Figure 5:**
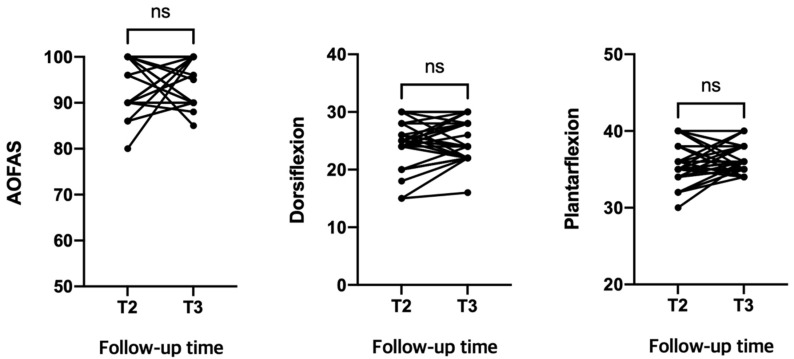
Functional outcomes before (T2) and after hardware removal (T3). Mean and standard deviation of AOFAS at T2 (94.3 ± 5.8) and T3 (95.9 ± 5.0); ankle dorsiflexion at T2 (24.3 ± 3.8 degrees) and T3 (25.7 ± 3.5 degrees); ankle plantarflexion at T2 (35.7 ± 2.7 degrees) and T3 (36.2 ± 1.8 degrees).

Clinically, all patients described the resolution of symptoms related to painful hardware at the final follow-up. The AOFAS score and ankle range of motion did not show any significant difference after SB removal (Figure 5). None of the patients in the study cohort suffered complications related to hardware removal.

## 4. Discussion

Despite the significant reported rate of SB removal, the clinical and radiographic implications are poorly investigated, which has created uncertainty in making surgical decisions in patients with painful hardware symptoms. Therefore, we sought to examine the changes after SB removal in patients presenting with symptomatic hardware. The current study results show that SB removal one year after initial fixation was not associated with recurrent diastasis while alleviating pain and discomfort associated with hardware. There were no complications after SB removal. 

One of the major advantages of adopting SB over screw fixation is to negate the need for routine hardware removal. In screw fixation, it has been suggested that recurrent diastasis may be attributed to the early removal of hardware before the consolidation of syndesmotic ligaments. As the time frame needed for syndesmotic ligament healing remains unclear, SB is an advantageous method of fixation because it allows for continued weightbearing and exercise during the suspected healing period without removing the hardware. Due to this advantage, some studies have reported a shorter time to return to work and sports activity in SB, compared to screw fixation [20,25]. However, contrary to its original design concept, a considerably high SB removal rate has been reported (Table 2) [11,12,13,14,15,20,26,27,28,29,30,31,32,33,34,35,36]. The causes described in the literature include infection, osteomyelitis, osteolysis, and most notably, irritation from the hardware after fracture healing.

As the SB consists of a metal oblong button and fiberwire, the resulting protrusion of the construct can cause irritation, which is the most common cause of removal. Furthermore, in the case of simultaneous ankle fractures, the removal of the fracture hardware often entails the removal of the SB, as the SB is often engaged within the lateral plate [11]. However, despite its considerable removal rate, this removal was not performed under an evidence-based approach as the subsequent outcomes following SB removal have been unknown. Given this context, the role of the SB in resisting diastasis after fracture healing remained a question.

With unchanged functional outcomes before and after removal, the SB does not appear to limit the range of ankle motion by non-physiologically tightening the tibiofibular joint one year after initial fixation. Furthermore, SB removal did not result in any radiographic changes after removal both in radiographic and CT analysis, suggesting that the tibiofibular relationship is already consolidated at the time of removal. Thus, it can be assumed that the SB’s ability to resist diastasis was no longer present at the time of removal in the current study, which favors the removal of symptomatic hardware after fracture healing.

In previous CT analyses investigating the impact of screw removal on syndesmosis integrity, removal of a rigid fixation system yielded a reduction in patients with a malreduced syndesmosis [6,7]. While SB removal in the current study did not demonstrate such findings, we observed spontaneous reduction of malreduced syndesmoses, while the SB was retained, which was previously only described in an in vitro study [37]. The efficacy of malreduction correction with SB has been previously demonstrated in anatomic research by Westermann et al. In their research, the authors concluded that the SB was superior to screw fixation in correcting deliberate malreduction of the syndesmosis [37].

**Figure 6:**
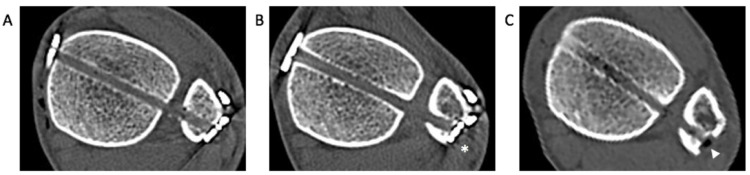
Sequential axial images of computed tomography showing cortical lysis of the fibula following suture button (SB) fixation: (**A**) immediate postoperative image showing intact fibular cortex facing the oblong button of the SB; (**B**) a year after initial fixation, medial translation of the oblong button is noticed (asterisk) with a mild widening of the tibiofibular space; (**C**) after SB removal, definite cortical lysis of the fibula is observed (white arrowhead).

The mechanism of how spontaneous reduction with retained SB occurs is not fully understood. Westermann and colleagues postulated that oblique positioning of the suture thread within the tunnel allows for micromotion of the fibula relative to the tibia incisura [37]. Peterson et al. described this micromotion using the term “creep,” resulting from elongation of the suture due to repetitive weightbearing [38]. In our study cohort, we found evidence of cortical lysis on CT imaging following SB removal in several patients (Figure 6). We hypothesize that this may have allowed some degree of motion within the tibiofibular joint, making the implant more forgiving to perioperative malreduction. Furthermore, our findings may partially explain a relatively lower malreduction rate of SB fixation, compared to screw fixation [39].

This study has several limitations. First, as the study cohort mainly consisted of relatively active young male patients admitted to military hospitals, this study is subject to selection bias. Second, this was a retrospective investigation with a relatively small number of patients who underwent SB removal. Additionally, the current study was a cases series without a control group for comparison. Nonetheless, SB removal is not a routine procedure, rendering this study the first and largest series to date on the outcomes following SB removal. As a major cause of SB removal is skin irritation, a surgical technique to minimize skin irritation should be further emphasized [40]. Although a recent knotless SB system has been introduced to minimize skin irritation, we believe our results are meaningful because asymptomatic SB is still being removed together with the lateral plate at the time of fracture hardware removal [11]. Third, the follow-up period after SB removal was relatively short. However, a previous study by Song et al. observed a reduction of malreduced syndesmosis one month after screw removal [6]. Drawing from their findings, we deemed an average of five months as adequate to observe changes on both plain radiograph and CT imaging. Fourth, the functional outcome was assessed with an AOFAS score, which is currently believed not to be a validated measure. However, its use has been demonstrated in numerous outcome studies with a syndesmosis injury. Additionally, the patients included in the study cohort had their index encounter in 2007, and this scoring system was used throughout the study period to remain consistent in evaluating functional outcomes. Finally, we believe that weightbearing X-rays and weightbearing CT have the potential to assess reduction status more accurately. In our study, we opted for non-weightbearing radiographs for the purpose of consistently comparing each of the three follow-up periods.

## 5. Conclusions

Our findings suggest that SB removal at an average of one year following initial syndesmotic fixation in the young, healthy cohort alleviates pain and discomfort without negatively impacting syndesmosis reduction. Therefore, we believe the current study results can be considered a helpful resource in managing patients with SB-related symptomatic hardware. In addition, we observed spontaneous correction of malreduction during the convalescent period, which was previously highlighted only by in vitro anatomic experiments.

## Figures and Tables

**Table 1:** Patient characteristics.

Patient No.	Age (Years)	Sex	Fracture Pattern	SB Removal (Months)	FU (Months)
1	25	M	SER (LM, PM)	12	15
2	26	M	Isolated	13	16
3	24	M	SER (LM)	8	12
4	25	M	Maisonneuve	12	15
5	25	M	SER (LM)	12	15
6	24	M	Maisonneuve (MM)	12	15
7	27	M	PER (LM)	12	18
8	22	M	PER (LM, MM, PM)	12	15
9	24	M	PER (LM)	12	15
10	22	M	SER (LM)	12	15
11	24	M	Maisonneuve (MM)	14	18
12	28	M	PER (LM)	12	15
13	22	M	SER (LM)	12	15
14	25	M	SER (LM)	12	24
15	24	M	PER (LM)	12	15
16	33	M	Maisonneuve	19	36
17	25	M	SER (LM)	12	15
18	26	M	SER (LM)	13	20
19	23	M	PER PM	12	15
20	26	M	SER (LM)	10	16
21	21	M	SER (LM)	12	25
22	26	M	SER (LM)	12	24
23	22	M	PER (LM, MM, PM)	12	24
24	21	M	SER (LM)	13	16
25	22	M	SER (LM)	14	18
26	23	M	SER (LM)	14	18
27	21	M	SER (LM)	10	19
28	20	M	Isolated	13	22
29	23	M	SER (LM)	16	19
30	21	M	SER (LM, PM)	11	20
31	22	M	PER (LM, MM, PM)	13	18
32	35	M	Isolated	7	17
33	35	M	isolated	12	16

Abbreviations: SER, supination external rotation fracture in Lauge–Hansen classification; PER, pronation external rotation fracture in Lauge–Hansen classification; LM, lateral malleolar fracture; PM, posterior malleolar fracture; MM, medial malleolar fracture; SB, suture button; FU, follow-up.

**Table 2:** List of the literature on the removal of suture button after syndesmotic fixation.

Authors	No. of Patients with Removal/Total	Cause of Removal	Time at Implant Removal	Radiographic Outcome
McMurray et al. [15] (2007)	2/16 (12.5%)	Infection (1) Irritation (1)	NR	NR
Willmott et al. [12] (2009)	2/6 (33.3%)	Irritation	6 Mo/10 Mo	No diastasis
Treon et al. [31] (2009)	4/18 (22.2%)	Wound breakdown, knot prominence	NR	NR
Gadd et al. [14] (2009)	3/38 (7.9%)	Osteomyelitis (2) postoperative fall (1)	NR	NR
Coetzee and Ebeling [28] (2009)	1/12 (8.3%)	Infection	6 mon	NR
Qamar et al. [30] (2011)	1/16 (6.3%)	Irritation	NR	NR
DeGroot et al. [29] (2011)	6/24 (25%)	Irritation (4) Pain (1) Open fracture (1)	12~35 Mo (4) 12 weeks (1) 8 weeks (1)	No diastasis
Naqvi et al. [32] (2012)	3/49 (6.1%)	Infection (1) Infected sinus formation (1) Irritation (1)	NR 6 Mo 5 Mo	No diastasis
Rigby et al. [34] (2013)	4/37 (10.8%)	Irritation	NR	
Maemple et al. [36] (2014)	1/12 (8.3%)	Routine removal	NR	NR
Seyhan et al. [13] (2015)	6/15 (40%)	Irritation (4) Irritation (2)	NR (4) 8 Mo/12 Mo (2)	NR
Kortekangas et al. [35] (2015)	1/21 (4.8%)	Infection	6 weeks	NR
Laflamme et al. [20] (2015)	2/34 (5.9%)	Infection	14 weeks	No diastasis
Kocadal et al. [33] (2016)	1/26 (3.8%)	Irritation	NR	NR
Bondi et al. [27] (2016)	1/36 (2.8%)	Irritation	6 Mo	No diastasis
Anand et al. [26] (2017)	1/36 (2.8%)	Irritation	6 Mo	NR
Anderson et al. [11] (2018)	11/48 (22.9%)	Local discomfort from SB (6) and plate (5)	NR	No diastasis

Abbreviations: SB, suture button; Mo, month; NR, not reported.

## Data Availability

The datasets used and/or analyzed during the current study are available from the corresponding author on reasonable request.
